# Differential modulation of the cortical alpha rhythm and activation of distinct neural networks during tactile perception training by learners and non-learners

**DOI:** 10.3389/fnins.2025.1566615

**Published:** 2025-05-09

**Authors:** Kei Saito, Naofumi Otsuru, Kaito Tateishi, Ryuji Kurebayashi, Hideaki Onishi

**Affiliations:** ^1^Department of Physical Therapy, Niigata University of Health and Welfare, Niigata, Japan; ^2^Institute for Human Movement and Medical Sciences, Niigata University of Health and Welfare, Niigata, Japan

**Keywords:** alpha band, functional connectivity, perceptual learning, power spectral density, tactile orientation discrimination

## Abstract

**Background:**

The sensitivity and discrimination capacity of sensory systems can be improved by perceptual training. Most individuals demonstrate tactile perceptual learning, but with marked differences in efficiency. Here, we investigated the neural mechanisms underlying individual differences in tactile learning efficiency at the network level.

**Methods:**

Electroencephalographic (EEG) signals were recorded from 25 neurologically healthy participants at baseline, after one training session (50 trials) on the tactile grating orientation discrimination task (GOT), and again after four sessions of GOT training (200 training trials in total). Participants were then divided into low- and high-learning groups based on the post-training change in GOT threshold (sensitivity). Cortical alpha-band power, which is associated with sensory processing efficiency, was compared between baseline and post-training in low- and high-learning groups. Coherence analysis was also performed between EEG electrode pairs to reveal functional connectivity (FC) networks associated with low and high learning.

**Results:**

In the high-learner group, alpha-band power spectral density (PSD) was significantly stronger post-training at the left central-parietal electrodes. In addition, FC in the alpha band was significantly strengthened within left frontal-parietal regions after training. In the low-learner group, post-training alpha-band PSD was significantly strengthened at the bilateral frontal-central electrodes, while FC in the alpha band did not change significantly compared to baseline.

**Conclusion:**

These results suggest that individual differences in tactile learning may result from the utilization of distinct neural networks.

## 1 Introduction

Tactile spatial discrimination ability varies widely across individuals but can be improved through training (Wong et al., 2013; Dempsey-Jones et al., 2021; Sakai et al., 2021). A more complete description of the underlying neural mechanisms is needed as this form of perceptual learning underlies important human skills, while functional recovery of tactile perception is essential for motor rehabilitation following neuropathological events such as stroke (Au-Yeung and Hui-Chan, 2009). Perceptual learning is defined as the process by which exposure to a specific perceptual stimulus, whether naturally or in a regulated training program, improves the sensitivity to that stimulus and the fine discrimination of similar stimuli within the same modality (Sagi, 2011; Wong et al., 2013; Cybulska-Klosowicz et al., 2020; Dempsey-Jones et al., 2021; Sakai et al., 2021). However, elucidation of the mechanisms underlying perceptual learning has been hampered by the marked heterogeneity in both baseline competence and training-induced improvement among study participants (Mukai et al., 2007; Muller-Gass et al., 2017; Sakai et al., 2021).

Endogenous neural oscillations in the 8–13 Hz (alpha) range enhance cortical information processing efficiency by inhibiting behaviorally irrelevant information (Klimesch et al., 2007; Rihs et al., 2007; Haegens et al., 2011; Jensen and Mazaheri, 2010; Jensen et al., 2014). Thus, alpha oscillations in the primary somatosensory cortex are critical for the processing of tactile information. Ai and Ro (2014) reported an inverted U-shaped relationship between the alpha oscillation power in primary somatosensory cortex and tactile perception, indicating that tactile perception is sharpest when these regional alpha oscillations are of optimal strength. Similarly, paired-pulse depression (PPD), which reflects inhibitory control processes as well as alpha oscillation power, is associated with tactile spatial discrimination performance (Lenz et al., 2012; Sasaki et al., 2023). Our previous study revealed weaker PPD in the somatosensory cortex of healthy young individuals showing superior tactile two-point discrimination performance (Sasaki et al., 2023). Collectively, these findings indicate that optimal cortical inhibition associated with alpha oscillations is essential for tactile spatial discrimination performance.

Therefore, differential activation of alpha oscillations in sensory cortex and associated networks may contribute to individual differences in perceptual learning. Muller-Gass et al. (2017) reported that alpha oscillations in cortical areas, including early visual areas, were more powerful in a high-learner group than in a low-learner group following perceptual training. Furthermore, these oscillations grew even stronger in the high-learner group as training progressed. Similar findings have been reported in the tactile domain. Brickwedde et al. (2019) also reported that perceptual learning was more effective when the baseline alpha oscillations were stronger in somatosensory regions. However, it is currently unclear how alpha oscillations in early somatosensory areas are related to interindividual variations in tactile perceptual learning. Functional connectivity (FC) strength between the early (primary) sensory cortex and other cortical regions may also contribute to the individual differences in perceptual learning. Mukai et al. (2007) found that the FC between early visual areas and the intraparietal sulcus/frontal eye field was enhanced by training on a visual discrimination task among a high-learner group, whereas the FC between the early visual areas and the fusiform gyrus was strengthened in a low-learner group.

Collectively, these results suggest that high and low learners engage distinct neural mechanisms. Thus, alpha oscillation power in the relevant early somatosensory area and the FC strength between this early somatosensory area and other cortical regions may be responsible for interindividual variations in tactile perceptual learning, although this remains to be conclusively demonstrated. Therefore, this study compared regional alpha oscillation power and FC of early somatosensory areas with other cortical regions between high and low perceptual learners to determine if individual differences in perceptual learning efficiency result from the engagement of distinct neural mechanisms.

## 2 Materials and methods

### 2.1 Participants

Twenty-nine right-handed males (mean ± standard deviation, 21.1 ± 0.5 years of age; range, 20–22 years) participated in a perceptual learning experiment using the tactile grating orientation discrimination task (GOT). In addition, 26 participants (20.7 ± 0.6 years of age; range, 20–22 years) were recruited for a control experiment to confirm that GOT performance changes represented a true training (perceptual learning) effect. Exclusion criteria for all participants included histories of neurological, cardiovascular, and psychiatric disorders, diabetes, the use of medications that potentially influence the central nervous system, factors known to alter tactile acuity such as calluses at the test site (right index finger), and past participation in perceptual learning experiments. The Edinburgh Handedness Inventory (Oldfield, 1971) was used to determine the dominant hand of each participant. This study was conducted in accordance with the Declaration of Helsinki and was approved by the ethics committee of Niigata University of Health and Welfare. All participants provided written informed consent. Three participants in the GOT trial were excluded due to large EEG artifacts, and one participant was excluded due to lack of peak alpha power at electrode site CP3. Thus, 25 participants were included in the final analyses.

### 2.2 Experimental design

**Figure 1:**
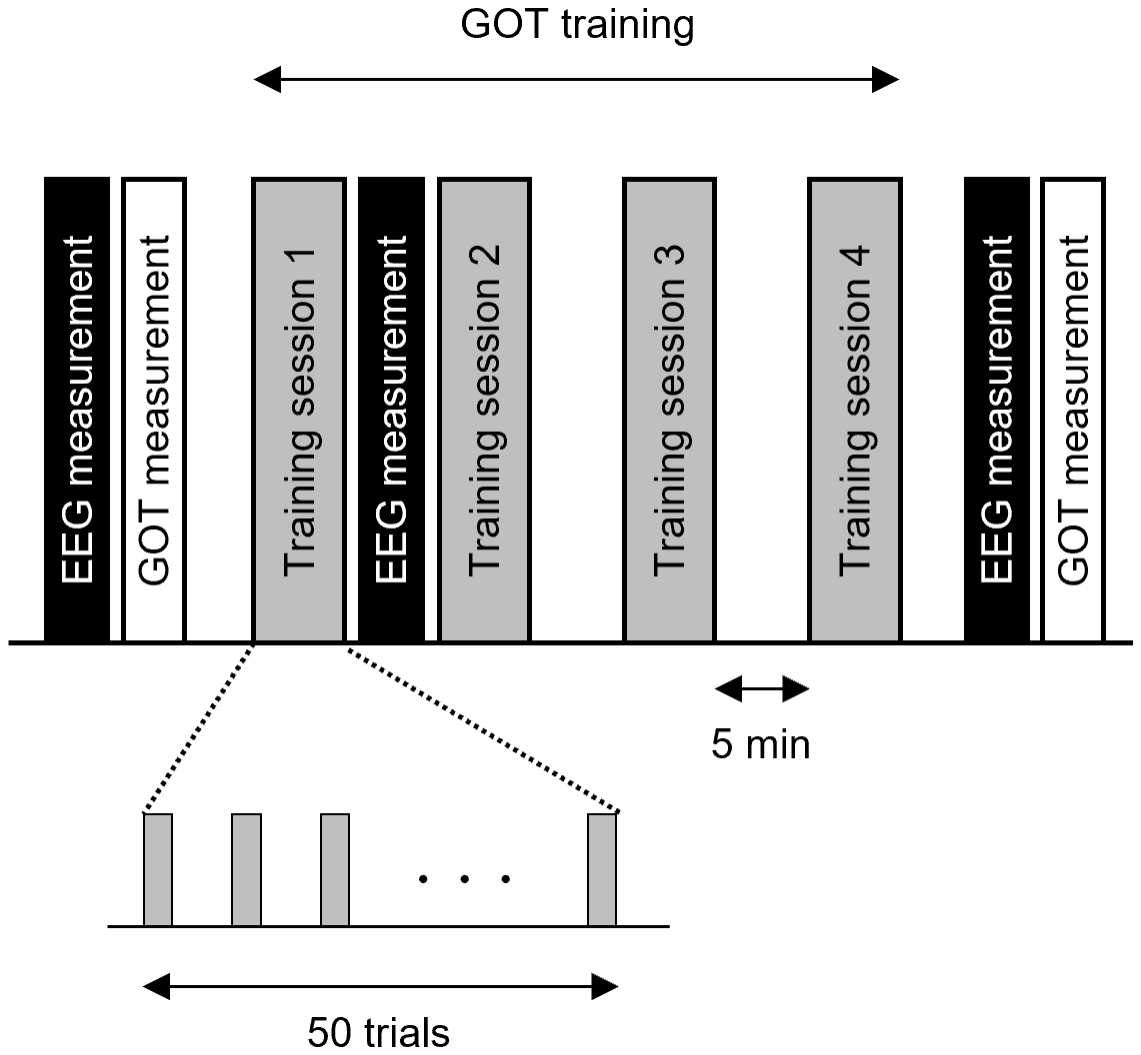
Schematic representation of the grating orientation discrimination task (GOT), tactile discrimination training procedure, and electroencephalogram (EEG) measures for the construction of tactile learning networks. Participants completed four training sessions of 50 GOT trials each. Performance on the GOT was assessed by measuring the orthogonal-parallel orientation discrimination threshold for grating size (0.35 mm to 3.0 mm) immediately before the first training session (baseline) and immediately after the final EEG session. The EEG was measured in the resting state with eyes open before the first training session (baseline), immediately after the first training session, and immediately after the fourth training session. Finally, the GOT threshold was remeasured and compared to the baseline value.

A schematic representation of the experiment is presented in Figure 1. A 5-min resting-state EEG recording was first acquired, followed by baseline assessment of tactile spatial acuity using the GOT as described in section 2.3. The participants were then trained on the GOT (200 trials split into four training sessions of 50 trials each) with auditory feedback on correct and incorrect responses. A second 5-min resting-state EEG recording was acquired between the first and second training sessions to examine the early neural changes associated with tactile perceptual learning. After all training sessions, a third 5-min resting-state EEG recording was acquired, followed by a post-training GOT test.

### 2.3 Stimuli

Tactile stimuli from eight hemispherical domes with different groove widths (3.0, 2.0, 1.5, 1.2, 1.0, 0.75, 0.5, and 0.35 mm) were delivered to the right index fingertip using a custom-made device that automatically controlled the upward and downward movements of the test dome (S-16026; Takei Scientific Instruments Co. Ltd., Niigata, Japan). The elevation speed was set to 20 mm/s and the tactile stimulation duration to 1 s according to the methods developed in our previous study (Saito et al., 2022). The hemispherical dome was elevated a further 1.5 mm after touching the right index fingertip to ensure delivery of the tactile stimulus (Saito et al., 2022).

### 2.4 Tactile spatial acuity assessment

The GOT is widely accepted as a valid measure of tactile spatial acuity (Sathian et al., 1997; Goldreich and Kanics, 2003; Ragert et al., 2008a; Ragert et al., 2008b). Tactile stimuli were delivered 16 times from each of the eight hemispherical domes to the right index finger, starting with the largest grating (3.0 mm) and ending with the smallest (0.35 mm), for a total of 16 × 8 = 128 dome presentations. The participants were asked to judge the grove orientation relative to the long axis of the finger (parallel or orthogonal) by pressing a button with the left index finger. The grating width was also plotted against the percentage of correct responses and fitted by logistic regression based on a generalized linear model to assess discrimination sensitivity. The GOT discrimination threshold was defined as the finest grating width yielding a 75% correct response rate (Ragert et al., 2008a; Ragert et al., 2008b; Fujimoto et al., 2014; Saito et al., 2022). The change in GOT discrimination threshold induced by training (ΔThreshold) was calculated as follows:


ΔThreshold=(Post training threshold−Baseline threshold)/Baseline threshold×100

The baseline threshold was measured immediately before the first training session and the post-training threshold was measured immediately after the final EEG measurement (Figure 1).

### 2.5 Training sessions

Participants completed four training sessions of 50 trials with 5-min breaks between each session as described previously (Wong et al., 2013; Sakai et al., 2021). One training session took approximately 7 min. On each trial, the GOT orientation (orthogonal or parallel) was random. Correct and incorrect responses were indicated by auditory feedback following the button press. For the first training session, the grating width nearest to each individual participant’s 76% discrimination threshold as measured before training was used. For subsequent training sessions, the grating width was selected according to each individual’s proportion of correct responses in the preceding training session. The grating width was reduced (finer) if the proportion of correct responses was >90%, remained unchanged if 60–90%, and increased (coarser) if <60%.

### 2.6 EEG data acquisition and preprocessing

All EEG recordings were acquired in the resting state with eyes open. Participants wore a 64-channel active electrode cap (EasyCAP, Brain Products GmbH, Germany) with 63 channels in the international 10–10 system arrangement. In addition, a reference electrode was placed at position FCz and a ground electrode at position FPz. The signal from each channel was sampled at 2,500 Hz for subsequent offline analyses. The bipolar electrocardiogram (ECG) and bipolar electrooculogram (EOG) were recorded simultaneously with the EEG to detect artifacts caused by heartbeats and eye movements. Electrode impedance was below 5 kΩ for all recordings retained.

The EEG data were preprocessed using Brainstorm software (Tadel et al., 2011). Noisy or otherwise unusable channel recordings were removed using Welch’s method for power spectral density (PSD) estimation with 2-s time windows and 50% overlap, resulting in the removal of 4.48 ± 2.12 channels per individual. Continuous EEG recordings were then resampled offline to 250 Hz, band-pass filtered between 1 and 50 Hz, notch filtered at 50 Hz to attenuate power line contamination, and re-referenced to the average of all electrodes for normalization. Signal space projection was used to remove cardiac and eye movement artifacts. In addition, we visually checked the EEG data for contamination by muscle activity and removed such recordings.

### 2.7 PSD analysis of the EEG recordings

The PSD of each artifact-free downsampled recording was re-estimated using Welch’s method with 2-s Hamming windows, overlapping by 50%, to examine changes in alpha-frequency power at each channel. Briefly, the absolute power of each electrode was log-transformed and the result averaged within the 8–13 Hz frequency band to obtain single-channel alpha rhythm power values.

### 2.8 Functional connectivity analysis

Alpha-band FC values between electrode pairs were estimated by imaginary coherence, which captures only the coherence that cannot be explained by volume conduction through the exclusion of zero- or *π*-phase-lag connectivity (Nolte et al., 2004), using Brainstorm software (Tadel et al., 2011). First, each 5-min time series was divided into 150 nonoverlapping 2-s segments and Fourier transformed. Given that the PSD in the alpha band at the central-parietal electrode CP3 increased after training in the high-level learning group, imaginary coherence was calculated between CP3 and other electrodes across different frequency bands. The imaginary coherence was then averaged for the total length of the selected recording within the alpha-band (8–13 Hz) to construct an FC network for each individual participant.

### 2.9 Statistical analysis

The Shapiro–Wilk test was performed to determine whether the GOT discrimination threshold and the change in the GOT discrimination threshold were normally distributed. The GOT discrimination threshold immediately after training was compared to the baseline value by a two-tailed paired-sample *t*-test. The association between the baseline GOT discrimination threshold and Δ threshold immediately after complete training was examined for all participants by calculating the Spearman’s rank correlation coefficients. A Cluster-based permutation test (two-tailed Student’s *t*-test, 10,000 randomizations, significance alpha level of 0.05) was performed to detect channels where perceptual learning significantly altered the alpha-band PSD and imaginary coherence within the alpha-band. Previous studies reported that changes in both alpha-band PSD in cortical areas, including early visual areas, and visual area-based FC induced by visual perception training differed between the high- and low-learner groups (Mukai et al., 2007; Muller-Gass et al., 2017). To examine the relationship between tactile learning efficacy and alpha-band oscillations in cortical areas, including the primary somatosensory cortex, as well as alpha-band FC based on the primary somatosensory cortex, participants were first stratified into high- and low-learner groups according to whether the relative change in GOT discrimination threshold after complete training (four sessions) was above or below the median change in GOT discrimination thresholds for all subjects and compared for alpha-band PSD and imaginary coherence within the alpha-band. The Shapiro–Wilk test was performed to determine whether the GOT discrimination threshold was normally distributed in the high- and low-learner groups. The GOT discrimination threshold immediately after the training sessions was first compared to the baseline in the high- and low-learner groups using a two-tailed paired-sample *t*-test. A cluster-based permutation test (two-tailed Student’s *t*-test, 10,000 randomizations, *α* = 0.05) was performed to detect channels where perceptual learning significantly altered the alpha-band PSD and the imaginary coherence within the alpha-band in each high- and low-level learner participant. Furthermore, a Monte Carlo permutation test (independent Student’s *t*-test with false discovery rate correction, 10,000 randomizations, *p* < 0.05) was used to compare baseline alpha-band PSD and imaginary coherence between high- and low-level learning groups. All statistical analyses of GOT discrimination threshold data were conducted using SPSS software (version 25, IBM Corp., Armonk, NY, United States), whereas EEG results were analyzed using Brainstorm software (Tadel et al., 2011). A *p* < 0.05 was considered statistically significant for all tests.

## 3 Results

### 3.1 Effect of tactile training on GOT performance

**Figure 2:**
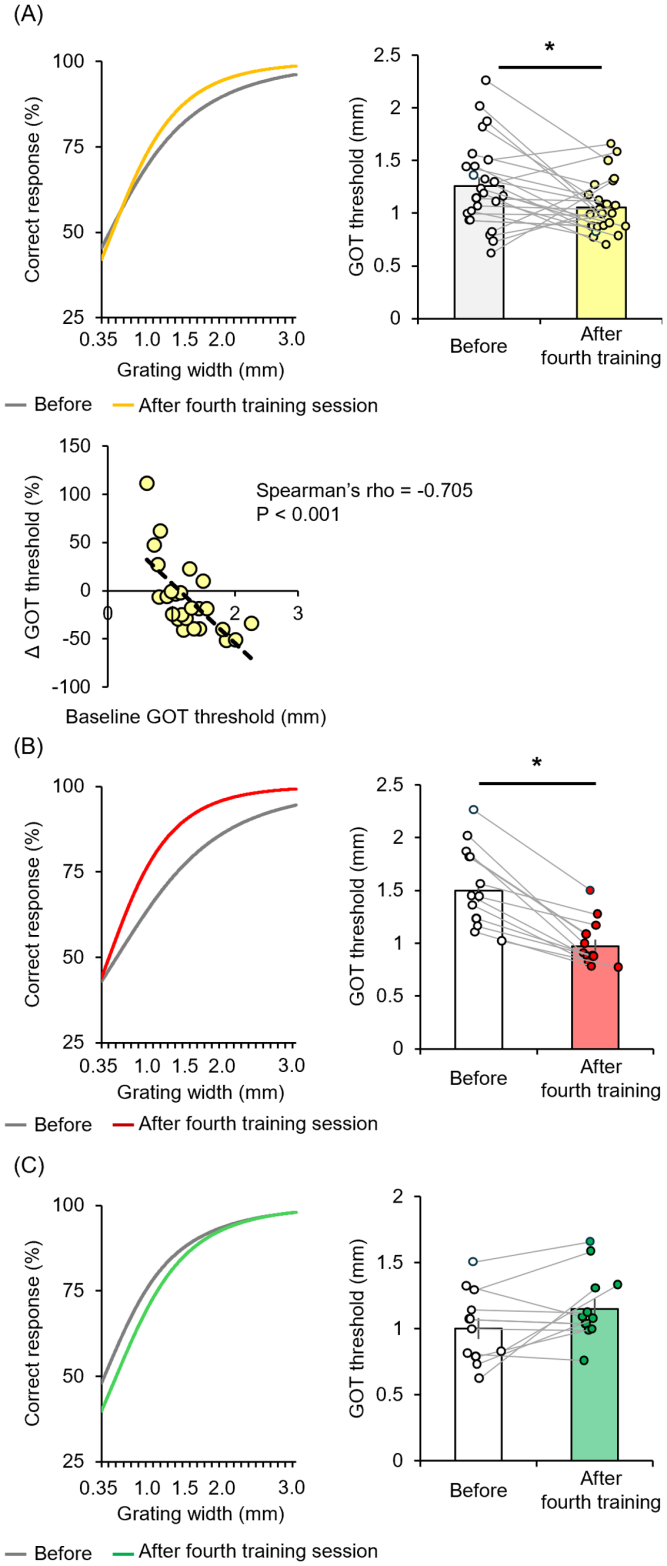
Tactile discrimination training reduced the GOT threshold only in a subset of participants (“high learners”) demonstrating larger baseline GOT thresholds. **(A)** In the entire cohort, four sessions of tactile training (post-training ×4) only modestly reduced the mean GOT threshold (white box: before training, yellow box: immediately after training). The relative change in GOT threshold from baseline (ΔGOT threshold) was negatively correlated with the absolute GOT threshold at baseline. **(B,C)** Training substantially reduced the GOT threshold in a subgroup of “high learner” participants (white box: before training, right red box: immediately after training) but not in a “low-learner” subgroup (white box: before training, green box: immediately after training). ^*^p < 0.05.

The mean GOT discrimination threshold for all participants was significantly reduced immediately after four tactile discrimination training sessions (50 trials per session for 200 total trials) [*t*_(24)_ = 2.335, *p* = 0.028 by two-tailed paired *t*-test, *r* = 0.43] (Figure 2A). To confirm that this decrease in threshold represented a true training effect, we conducted an additional experiment in which new participants (*n* = 26, 20.7 ± 0.6 years of age; range, 20–22 years) received the same stimuli but were requested to ignore the orientation, and found that perceptual learning did not occur [*t*_(25)_ = 0.917, *p* = 0.368, two-tailed paired *t*-test] (Supplementary Figure 1). A strong inverse correlation was observed between baseline GOT discrimination threshold and the relative change in GOT discrimination threshold (ΔGOT threshold) after training (Spearman’s rho = −0.705, *p* < 0.001). This implies that individuals with lower baseline discrimination ability exhibited a greater relative increase in performance.

However, the mean absolute ΔGOT discrimination threshold for the entire participant cohort was relatively modest, suggesting substantial heterogeneity in learning efficiency. Indeed, examination of individual participant results revealed a subgroup with no substantial reduction or even an increased threshold (Figure 2A). Moreover, when participants were stratified into a high-learner group (*n* = 13) with threshold changes below the median and a low-learner group (*n* = 12) with threshold changes above the median, only the former achieved a significant decrease in GOT discrimination threshold after the full training session [*t*_(12)_ = 7.085, *p* < 0.001, *r* = 0.90] (Figure 2B), whereas there was no significant difference among the low-learner group [*t*_(11)_ = −1.911, *p* = 0.082] (Figure 2C). We then compared EEG metrics between these groups to identify potential neural mechanisms underlying tactile discrimination learning.

### 3.2 Effect of perceptual training on the alpha-band PSD

**Figure 3:**
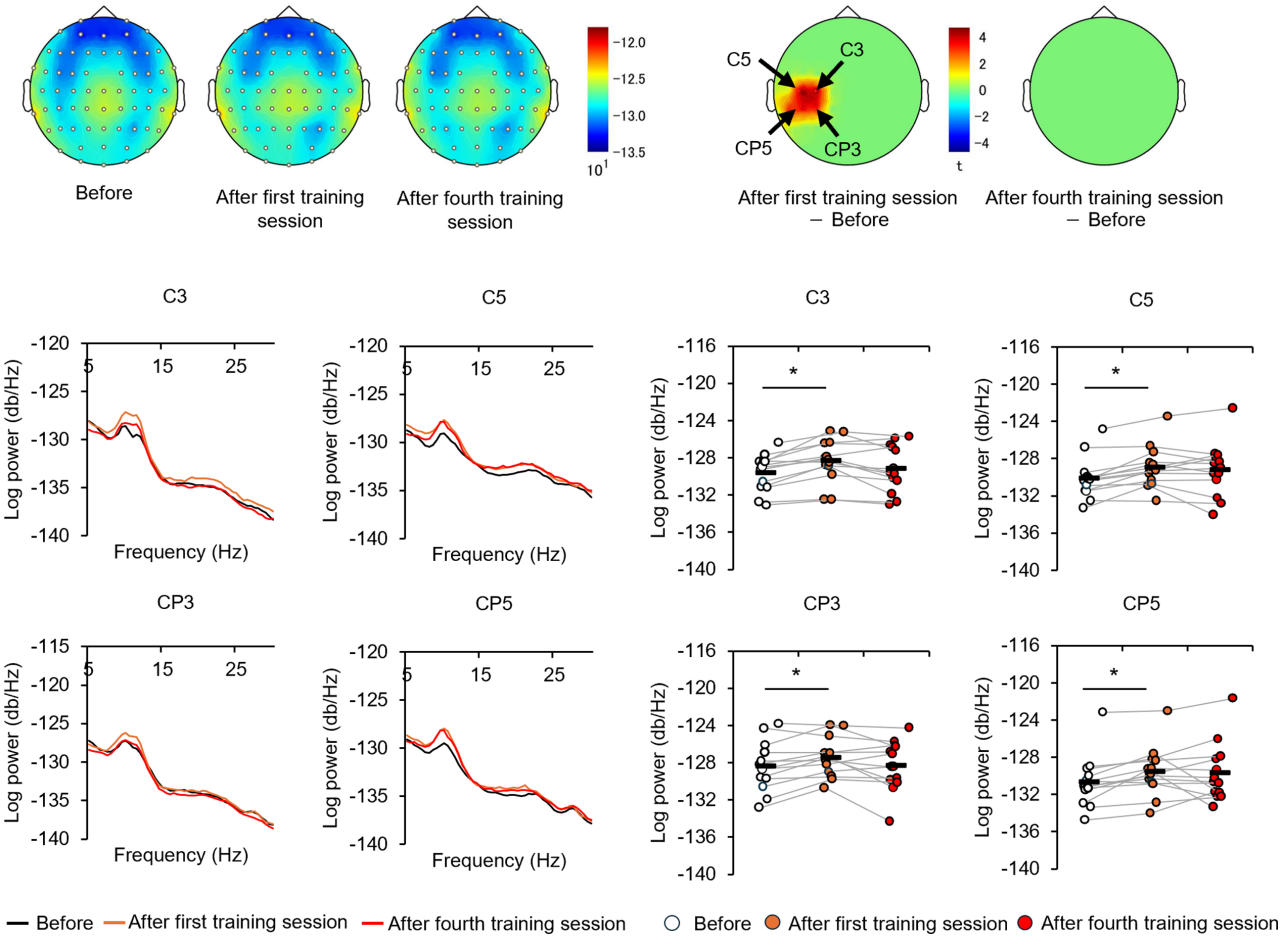
Tactile discrimination training influenced alpha-band power spectral density (PSD) in high-level learners. In this group, the first training session (post-training ×1) significantly increased alpha-band PSD at the left central-parietal electrodes. ^*^p < 0.05.

**Figure 4:**
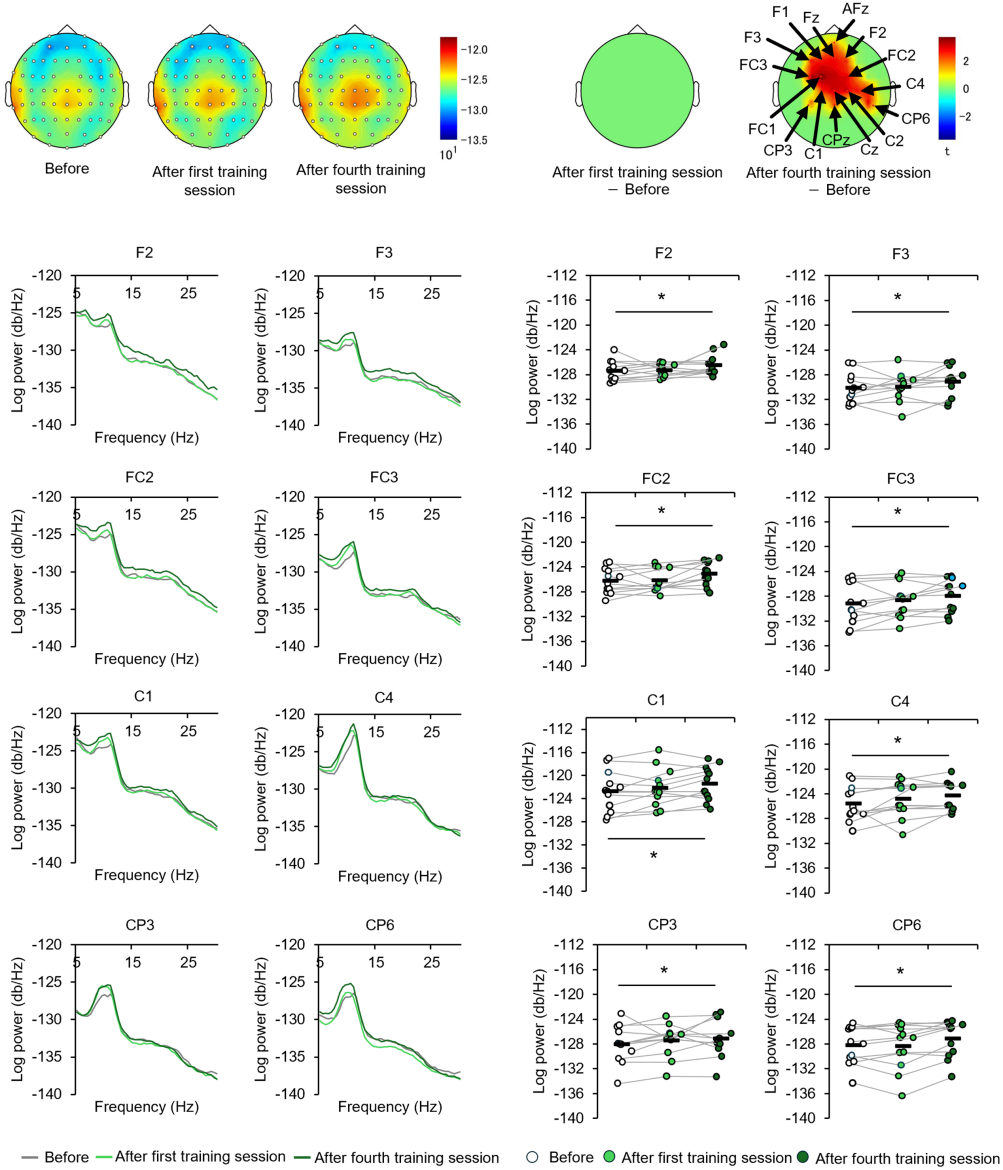
Tactile discrimination training influenced alpha-band PSD in low-level learners. No significant changes were observed after the first training session; however, after four training sessions (post-training ×4), alpha-band PSD was significantly stronger at bilateral frontal-central electrodes. ^*^p < 0.05.

**Figure 5:**
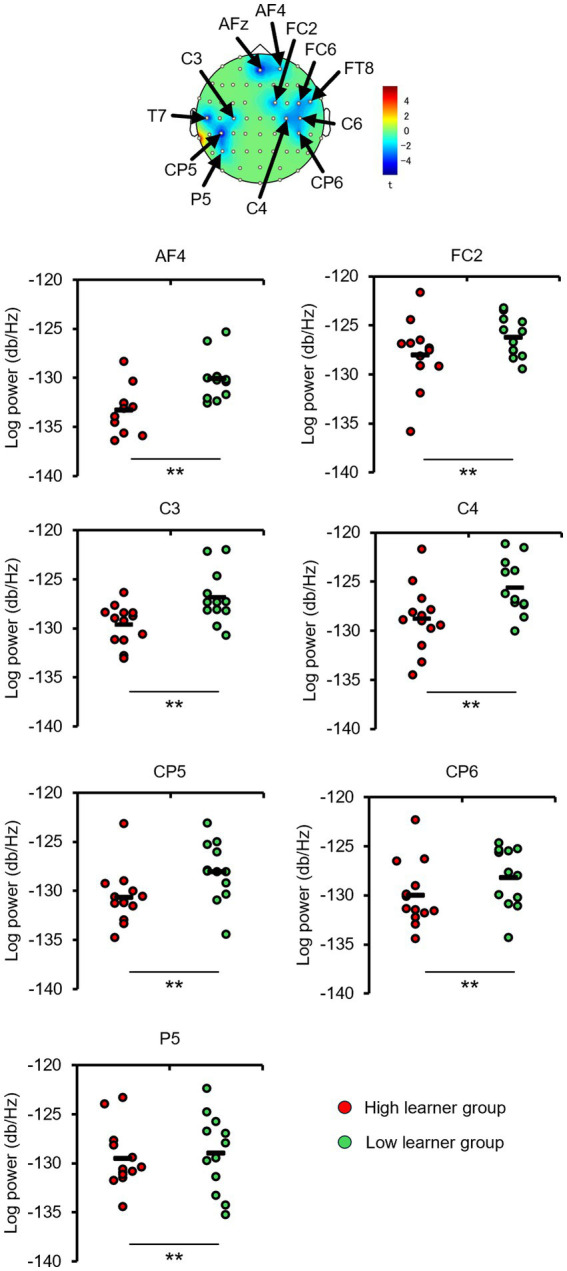
Baseline alpha-band PSD differs between high- and low-level learners. At the bilateral central-frontal electrodes, high-level learners exhibited significantly weaker baseline alpha-band PSD than low-level learners. ^**^p < 0.01.

The EEGs of the participants at baseline and after the first and fourth training sessions were then examined for differences to identify potential neural perceptual learning mechanisms. Mapping of the average alpha-band PSD for the entire cohort (Supplementary Figure 2) revealed a significant increase after the first training session (post-training ×1) compared with baseline at the left central-parietal electrode [CP3: *t*_(24)_ = 2.7618, *p* < 0.05; CP5: *t*_(24)_ = 3.3141, *p* < 0.05], corresponding to the left primary somatosensory cortex (Wolters et al., 2005). Significant increases were also observed at the left frontal-central electrode [FC3: *t*_(24)_ = 2.1307, *p* < 0.05] and the left central electrode [C3: *t*_(24)_ = 4.4436, *p* < 0.05; C5: *t*_(24)_ = 2.5915, *p* < 0.05]. Additionally, significant increases were noted at the bilateral central-parietal electrode [CP3: *t*_(24)_ = 2.1798, *p* < 0.05; CP5: *t*_(24)_ = 3.0403, *p* < 0.05; CP6: *t*_(24)_ = 2.0864, *p* < 0.05], the midline frontal electrode [Fz: *t*_(24)_ = 2.8685, *p* < 0.05], the bilateral frontal-central electrode [FC2: *t*_(24)_ = 2.0966, *p* < 0.05; FC3: *t*_(24)_ = 2.2427, *p* < 0.05], and the bilateral central electrode C3, C4, and C5 [C3: *t*_(24)_ = 2.7593, *p* < 0.05; C4: *t*_(24)_ = 3.4387, *p* < 0.05; C5: *t*_(24)_ = 2.4286, *p* < 0.05]. In the high-level learning group, the alpha-band PSD was also significantly stronger than baseline after the first training session at the left central electrode [C3: *t*_(12)_ = 4.8650, *p* < 0.05; C5: *t*_(12)_ = 4.9776, *p* < 0.05] and left central-parietal electrode [CP3: *t*_(12)_ = 3.8446, *p* < 0.05; CP5: *t*_(12)_ = 3.8905, *p* < 0.05] (Figure 3). Conversely, no significant differences were observed after the fourth training session at electrode sites (*p* > 0.05). In the low-learner group, there were no significant changes in alpha power after the first training session (all *p* > 0.05) (Figure 4), consistent with the behavioral results. However, after the fourth training session, alpha-band PSD was significantly stronger at the midline electrode [Fz: *t*_(11)_ = 2.8516, *p* < 0.05], bilateral frontal electrode [F2: *t*_(11)_ = 2.5684, *p* < 0.05; F3: *t*_(11)_ = 2.8313, *p* < 0.05], bilateral frontal-central electrode [FC1: *t*_(11)_ = 3.7519, *p* < 0.05; FC2: *t*_(11)_ = 3.0270, *p* < 0.05; FC3: *t*_(11)_ = 3.7304, *p* < 0.05], midline and bilateral central electrodes [Cz: *t*_(11)_ = 3.2830, *p* < 0.05; C1: *t*_(11)_ = 2.7611, *p* < 0.05; C2: *t*_(11)_ = 3.3238, *p* < 0.05], and midline and bilateral central-parietal electrode [CPz: *t*_(11)_ = 2.3379, *p* < 0.05; CP3: *t*_(11)_ = 2.2423, *p* < 0.05; CP6: *t*_(11)_ = 3.2369, *p* < 0.05]. Conversely, baseline alpha-band PSD was significantly weaker in the high-level learning group than in the low-level learning group at bilateral central electrode [C3: *t*_(23)_ = −2.9343, *p* < 0.01; C4: *t*_(23)_ = −3.5736, *p* < 0.01; C6: *t*_(23)_ = −2.9355, *p* < 0.01] and bilateral central-parietal electrode [CP5: *t*_(11)_ = −5.7702, *p* < 0.01; CP6: *t*_(11)_ = −3.2202, *p* < 0.01] (Figure 5). Overall, the group differences in baseline alpha-band PSD at the bilateral central and bilateral central-parietal regions, along with differential changes in alpha-band PSD at the left central and left central-parietal electrodes after the first training session, suggest unique perceptual responses and distinct learning mechanisms.

### 3.3 Effect of perceptual training on the FC

To examine whether these regional differences in alpha-band PSD between high- and low-efficiency learners reflect the activation of distinct cortical processing networks, CP3-based FC and subtraction connectivity networks were constructed among EEG channels using imaginary coherence analysis (Supplementary Figure 3). There were no significant changes in the alpha-band FC of the C3-based network after training in the entire cohort (*p* > 0.05).

**Figure 6:**
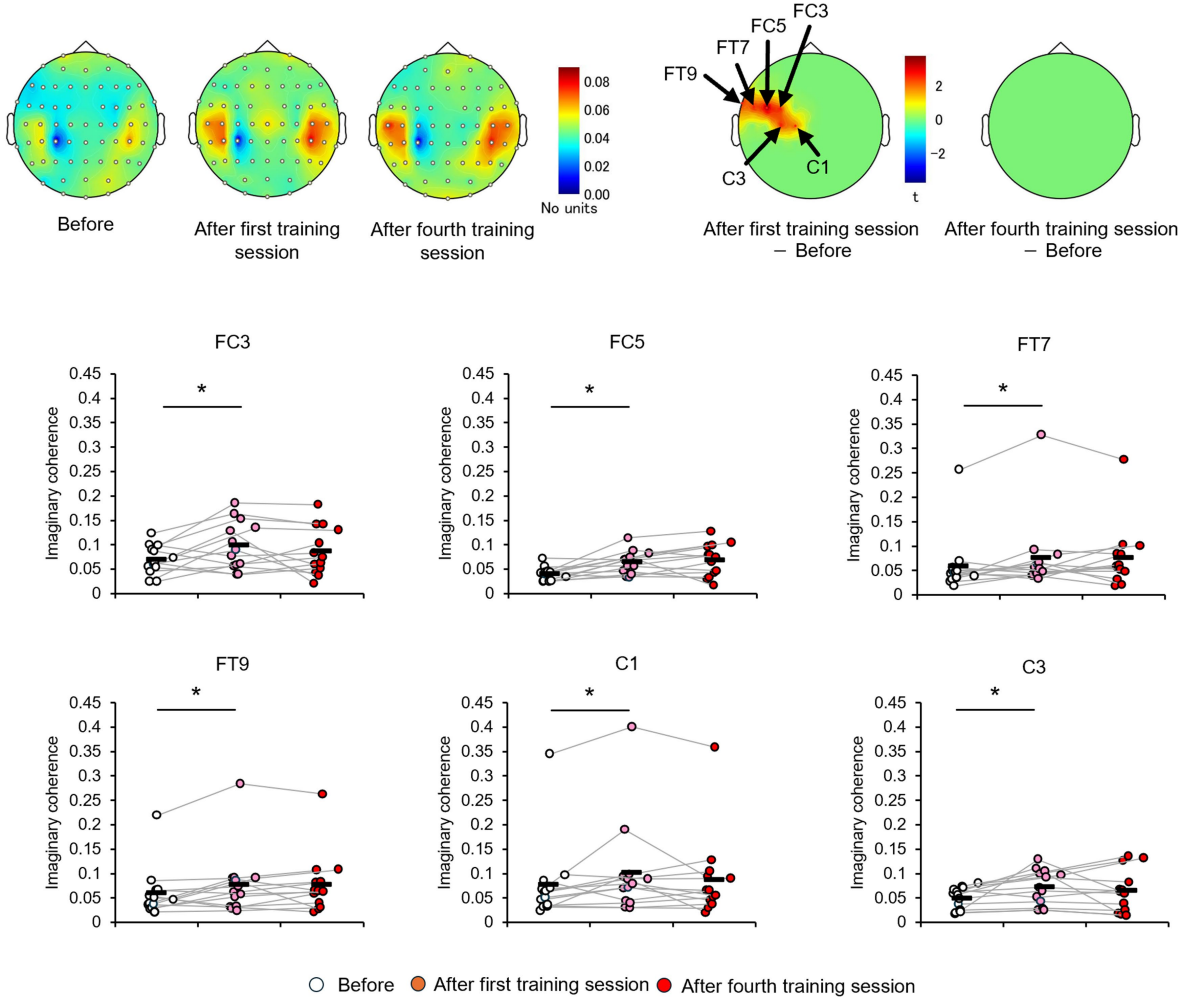
Tactile discrimination training, based on differentially modulated CP3-based network functional connectivity (FC) within the alpha-band, was assessed in the high-level learning group using imaginary coherence analysis. In this group, the tactile discrimination training significantly strengthened alpha-band FC between the CP3 electrode site and electrodes C1, C3, FC3, FC5, FT7, and FT9 compared with the baseline. ^*^p < 0.05.

**Figure 7:**
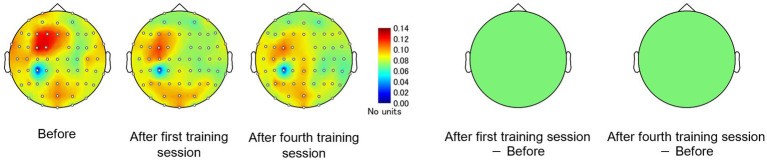
Tactile discrimination training with differentially modulated CP3-based network FC in the alpha-band, estimated using imaginary coherence analysis in the low-level learning group. In this group, alpha-band FC of the C3-based network remained unchanged after training.

**Figure 8:**
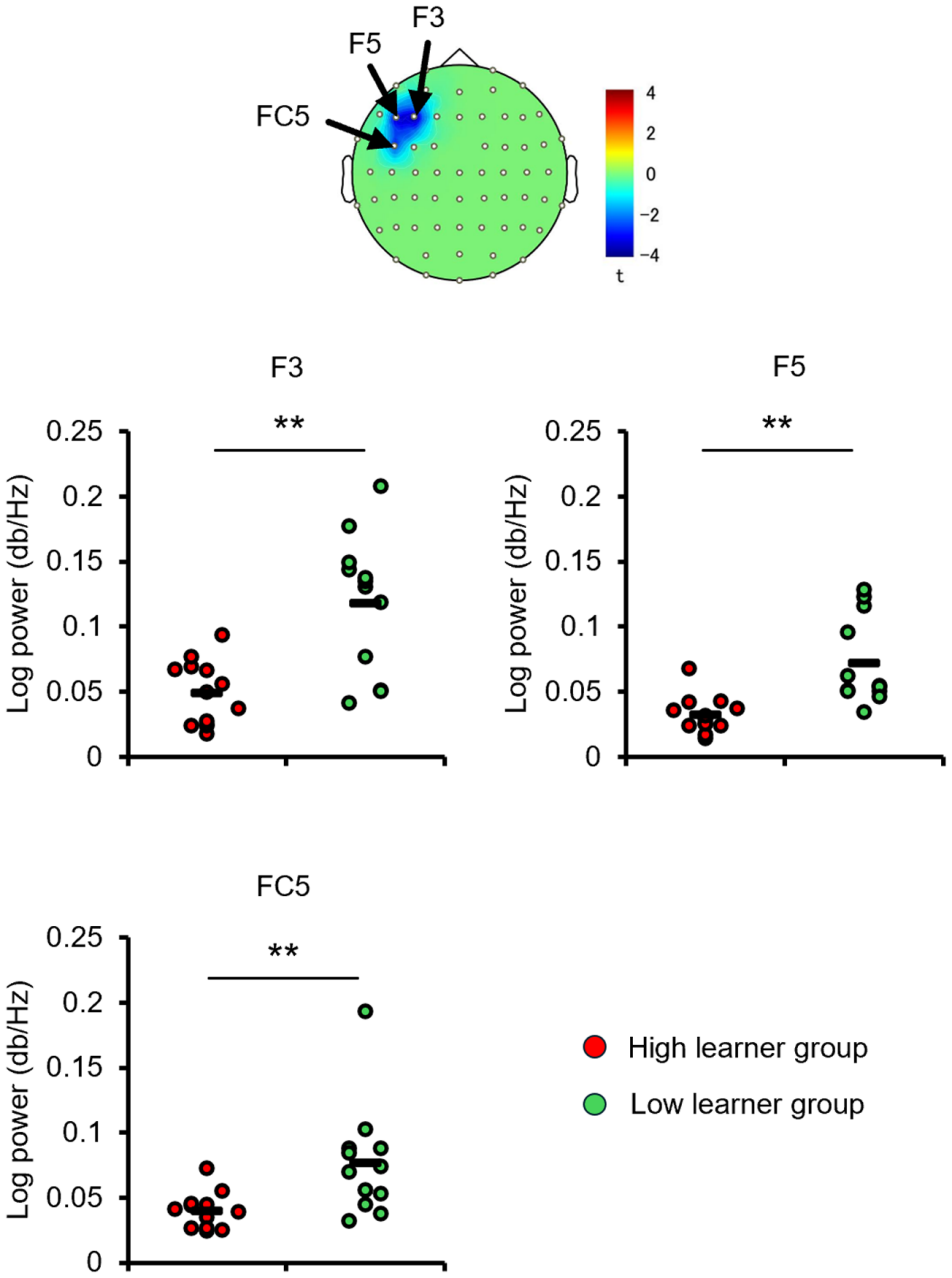
The baseline CP3-based network functional connectivity (FC) in the alpha-band differs between the high- and low-level learners. Baseline alpha-band FC between the CP3 electrode site and electrodes F3, F5, and FC5 was significantly weaker in the high-level learner group than the low-level learner group. ^**^p < 0.01.

Consistent with the behavioral results and alpha-band topology, these CP3-based FC patterns differed between the high- and low-learner groups. In the high-level learner group, alpha-band FC was significantly stronger after training (Figure 6), particularly in the left frontal-central regions, including CP3-FC3, after the first training session [*t*_(12)_ = 2.5512, *p* < 0.05]. Conversely, the low-level learning group showed no significant changes in alpha-band FC (all *p* > 0.05) (Figure 7). In addition to post-training FC, differences were detected in baseline FC. It was significantly weaker in the high-level learning group than in the low-level learning group, primarily in the left frontal-parietal and left central-parietal regions, including CP3-F3 [*t*_(23)_ = −4.2732, *p* < 0.01], CP3-F5 [*t*_(23)_ = −3.9673, *p* < 0.01], and CP3-FC5 [*t*_(23)_ = −2.4076, *p* < 0.05] (Figure 8). These results suggest that individual differences in tactile learning capacity may be linked to baseline and training-induced differences in alpha-band power and FC strength within networks spanning the left central-parietal, left frontal, and left frontal-central regions.

## 4 Discussion

This study presents evidence that efficient tactile perceptual learning is dependent on both low basal alpha-band power in the left central-parietal cortex and training-dependent strengthening of alpha-band FC in the frontal-parietal region. First, alpha oscillations in the left somatosensory cortex (reflected by EEG activity at electrode CP3) were substantially strengthened by a single tactile discrimination training session among participants demonstrating significant post-training improvement in the GOT (termed the high-learner group) but not among participants showing little GOT performance improvement after training (the low-learner group). Second, in the high-level learning group, baseline alpha-band FC between the left somatosensory and left frontal-central cortices was weaker but significantly strengthened by tactile discrimination training. Third, in the low-level learning group, baseline alpha oscillations were stronger in the right frontal-central cortex and further increased after four training sessions. Overall, these findings indicate that neural networks involved in tactile discrimination learning vary by learning ability and that this ability is closely linked to alpha-band power in neural networks spanning the parietal and frontal-central cortices.

### 4.1 Alpha oscillations involved in tactile perceptual learning in the high-learner group

The increase in alpha oscillation strength within the left somatosensory cortex (electrode CP3) after a single tactile discrimination training session (50 trials) among the high-learner group but not the low-learner group is consistent with our hypothesis that alpha oscillations in early somatosensory cortex are critical for tactile information processing. Similarly, Muller-Gass et al. (2017) reported that a visual discrimination training task increased the alpha oscillation strength in early visual areas only among high-learner participants. The primary somatosensory cortex encodes tactile information from peripheral sensory receptors (Salinas et al., 2000), and alpha oscillations modulate information processing in this and other brain regions by suppressing task-irrelevant information (Klimesch et al., 2007; Haegens et al., 2011; Jensen and Mazaheri, 2010; Jensen et al., 2014). However, the baseline alpha-band PSD in the high-learner group appears to differ between touch and vision modalities. The analyses presented here showing that weak baseline alpha-band PSD was subsequently strengthened by training suggest that inhibitory control in the left somatosensory cortex is enhanced by training on the tactile GOT using the right index finger, while Muller-Gass et al. (2017) found that only strong baseline alpha oscillations in early visual areas were subsequently strengthened by training. Several studies also have similarly reported an association between weaker baseline alpha oscillations and superior perceptual learning. Further, we recently reported that alpha-frequency transcranial alternating current stimulation (α-tACS) over primary somatosensory cortex improved tactile orientation discrimination in participants with weak baseline alpha oscillations but not in those with strong baseline alpha oscillations (Saito et al., 2021). Collectively, these findings suggest that perceptual learning is most efficient when alpha oscillations are relatively weak prior to perceptual training. In addition, alpha oscillations in the left somatosensory cortex were increased after only a single training session in the high-learner group, suggesting that weaker baseline alpha oscillations promote more efficient perceptual learning. This notion is consistent with the report by Neuling et al. (2013), who found that α-tACS was more effective for increasing alpha oscillation strength in participants with weaker baseline alpha oscillations. We suggest that these low basal alpha oscillations are sufficient for extraction of task-relevant sensory information and learning-induced neuroplastic changes, while large oscillations may inhibit activity too strongly for efficient perceptual learning.

### 4.2 Alpha-band neural connectivity involved in tactile perceptual learning among high-level learners

Our other core finding that alpha-band neural connectivity between the left somatosensory and left frontal-central cortices was weaker at baseline but strengthened by one training session in the high-learning group is also consistent with our hypothesis and a previous study reporting that FC between early visual areas and parietal/frontal regions was strengthened by visual discrimination training only in a high-learner group (Mukai et al., 2007). These results indicate that the FC between the early sensory and frontal cortices, such as the premotor cortex, is critical for perceptual learning. For instance, Romo and De Lafuente (2013) reported that the premotor cortex was activated after the primary secondary somatosensory cortices and posterior parietal cortex during a tactile discrimination task. Thus, we suggest that the decision-making network for discriminating tactile orientation was strengthened by training these high learners.

### 4.3 Alpha oscillations and alpha-band neural connectivity involved in tactile perceptual learning among low-level learners

Unlike high-level learners, who showed strengthened alpha-band neural connectivity between the left somatosensory and frontal cortices after training, the low-level learning group exhibited no change in FC strength following four training sessions. As stated, alpha oscillations were initially stronger in right frontal-central brain regions, and unexpectedly, alpha oscillations in bilateral frontal and bilateral frontal-central brain regions were further strengthened by four training sessions in the low-learner group. The stronger alpha-band power in the right frontal-central brain regions at baseline and post-training in the low-level learning group indicates that training impaired the decision-making regions involved in discriminating tactile orientation.

### 4.4 Differential modulation of alpha oscillations and alpha-band neural connectivity in tactile perceptual learning among high- and low-level learners

This study shows that neural networks involved in tactile discrimination learning differ based on an individual’s learning ability. Consistent with Mukai et al. (2007), who reported distinct networks for visual discrimination depending on learning ability, our findings indicate similar mechanisms in tactile learning. High-level learners had lower baseline tactile orientation discrimination ability, likely due to a reduced capacity to suppress task-irrelevant information in the primary somatosensory cortex and weaker neural networks associated with perceptual decision-making. However, tactile discrimination training improved their ability to suppress task-irrelevant information and strengthened the neural networks, ultimately improving tactile orientation discrimination. Conversely, low-level learners had higher baseline tactile orientation discrimination, attributable to a stronger ability to suppress task-irrelevant information and stronger neural network involvement in perceptual decision-making. However, tactile discrimination training may have excessively enhanced this suppression not only in the primary somatosensory cortex but also in frontal lobe regions involved in perceptual decision-making. This resulted in the suppression of task-irrelevant information necessary for tactile orientation discrimination, which prevented improvement in their tactile orientation discrimination performance.

### 4.5 Limitations

This study has several limitations. First, the small number of participants limited the statistical power. In addition, the small number of participants limits grouping by training effect to two groups (high- or low-learner group), which could result in participants being in opposite groups. Second, GOT performance was measured only at baseline and immediately after the final training session and EEG recordings, so the long-term effects of training on neural network activity and tactile discrimination are still unknown. Finally, we used a mass training protocol, while spaced training conducted over multiple days is frequently more effective and may result in distinct performance and neurological changes.

## 5 Conclusion

Training-induced strengthening of alpha-band power in the somatosensory cortex and the FC between the somatosensory and frontal-central cortices is essential for tactile learning. Furthermore, individual differences in tactile learning efficiency are associated with the activation of distinct neural networks operating in the alpha-frequency band.

## Data Availability

The raw data supporting the conclusions of this article will be made available by the authors, without undue reservation.
