# Supplementary material for: Differential modulation of the cortical alpha rhythm and activation of distinct neural networks during tactile perception training by learners and non-learners
Source: Front Neurosci. 2025 May 9;19:1566615. doi: 10.3389/fnins.2025.1566615 (PMC12098483; doi:10.3389/fnins.2025.1566615)
Supplement: Supplementary file 1 [file Data_Sheet_1.docx]

Supplementary Material

**Supplementary Results**


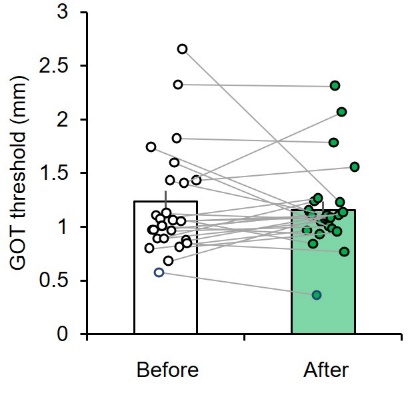


**Supplementary Figure 1.** Tactile stimulation without perceptual judgment fails to induce perceptual learning of the grating orientation discrimination task (GOT) as evidenced by the unchanged GOT threshold. Tactile stimuli (200 in 4 sessions) were delivered to the right index finger using a GOT hemispherical dome with grating width nearest to each participant’s 76% discrimination threshold measured prior to training trials. The participants were asked not to pay attention to the tactile stimuli and not to judge the dome orientation (parallel or orthogonal) relative to the long axis of the finger. The GOT discrimination threshold, defined as the grating width that permits 75% correct responses, was measured immediately before (Baseline) and immediately after this stimulation protocol (Post-training). There was no significant change in the threshold (t_(25)_ = 0.917, *p* = 0.368 by two-tailed paired t-test).


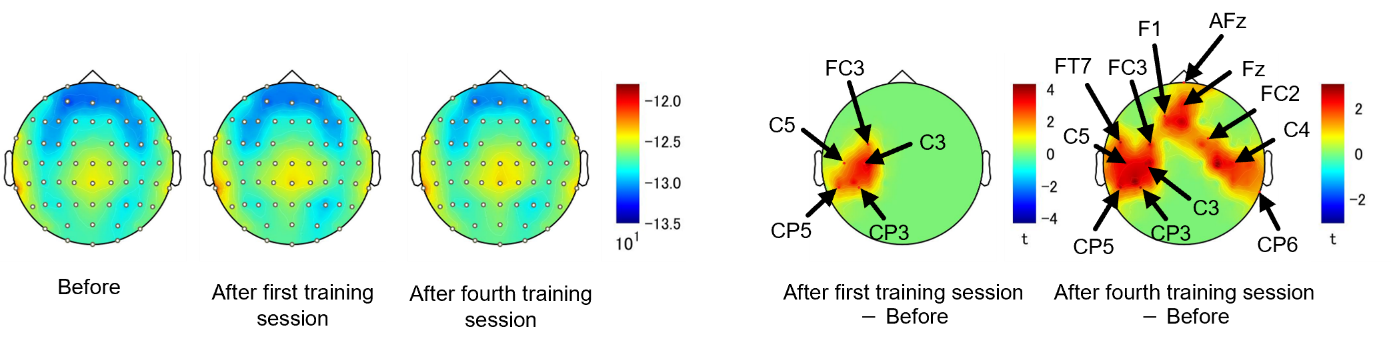


**Supplementary Figure 2.** Effect of tactile discrimination training on alpha-band power spectral density (PSD) across all participants. After the first training sessions (Post-training ×1), alpha-band PSD significantly increased at the left central–parietal and left frontal-central electrodes. Following the fourth training session, alpha-band PSD increased at right central-parietal and midline frontal electrodes.


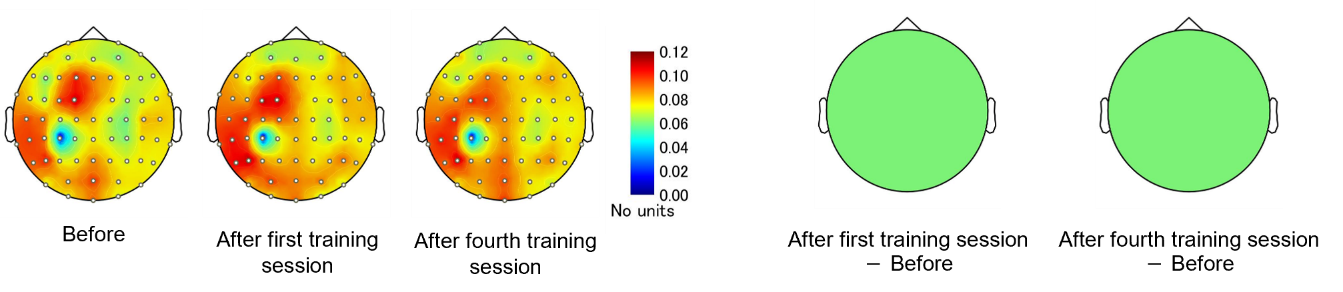


**Supplementary Figure 3.** Tactile discrimination training did not significantly affect functional connectivity based on somatosensory cortex (CP3 electrode).
